# Use of Exclusive Enteral Formula Diet as Adjunctive Therapy for Treatment of a Crohn’s Disease Flare

**DOI:** 10.1093/crocol/otaa006

**Published:** 2020-02-13

**Authors:** Levi M Teigen, Abigail J Johnson, Eugenia Shmidt, Byron P Vaughn

**Affiliations:** Department of Medicine, Division of Gastroenterology, Hepatology, and Nutrition, University of Minnesota, Minneapolis, Minnesota, USA; BioTechnology Institute, University of Minnesota, St. Paul, Minnesota, USA; Department of Medicine, Division of Gastroenterology, Hepatology, and Nutrition, University of Minnesota, Minneapolis, Minnesota, USA; Department of Medicine, Division of Gastroenterology, Hepatology, and Nutrition, University of Minnesota, Minneapolis, Minnesota, USA

**Keywords:** Crohn’s disease, exclusive enteral nutrition, gut microbiome

## Abstract

**Introduction:**

We report the case of an adult patient who achieved remission of a Crohn’s disease flare after treatment with exclusive enteral nutrition as adjunctive therapy to medication.

**Case Report:**

A 46-year-old man with severe, stricturing Crohn’s presented for severe abdominal pain and weight loss; estimated Crohn’s Disease Activity Index score greater than 300. Antibiotics, vedolizumab, budesonide, and exclusive enteral nutrition diet were instituted. Approximately 30 days later, his Crohn’s Disease Activity Index score improved to 170.

**Discussion:**

This case illustrates the possible utility of an exclusive enteral formula diet as an adjunct to medication to induce remission of a Crohn’s disease flare.

## INTRODUCTION

Inflammatory bowel disease (IBD) is a chronic, relapsing-remitting inflammatory disease of the intestine that affects over 3 million people in the United States.^1^ Although the etiology of IBD is unknown, it is thought to arise from an aberrant immune response to the intestinal microbiota in a genetically susceptible host.^2^ Crohn’s disease (CD), one of the 2 main forms of IBD, is occasionally treated with exclusive enteral nutrition (EN) in pediatric populations.^3^ In pediatrics, exclusive EN is up to 80% effective at inducing remission of symptoms.^4^ However, in the adult population, there is a paucity of data on EN therapy and its use remains minimal and all but absent from adult CD treatment guidelines.^4,5^ Additionally, there is little information on how EN affects the intestinal microbiota.

Beyond its purported role in inducing remission, EN therapy can also play a critical role in maintaining nutrition status in individuals with active disease. In a recent study conducted by Casanova et al,^6^ disease activity was one of the primary predictors of malnutrition risk (odds ratio = 4.3; 95% confidence interval 2.2–8.2). Therefore, EN therapy has the potential to play a dual role as a specific nutrition intervention and also as an adjunctive therapy to help achieve disease remission in adult populations with CD. We report the case of a patient who achieved remission of a CD flare after treatment with exclusive EN using a semi-elemental formula (Peptamen 1.5) as adjunctive therapy and describe the microbiota changes associated with EN.

## CASE REPORT

A 46-year-old man with severe, stricturing Crohn’s ileocolitis diagnosed at age 28, with a history of multiple stricturoplasties and ileocolic resection presented to the emergency department for severe abdominal pain and weight loss (day 0). His prior medical therapies included azathioprine, infliximab, methotrexate, adalimumab, certolizumab, and natalizumab, which were all characterized by either adverse reactions or disease progression. His current medical therapy was methotrexate and ustekinumab. He underwent radiographic imaging (CT enterography) 7 days prior to this presentation, which indicated multiple areas of high-grade luminal narrowing with evidence of chronic and active inflammation, associated dilation of the proximal small bowel, and entero-enteric fistula formation. Urgent surgery was considered however given prior small bowel resection, there was a concern for short bowel syndrome with further surgery. The estimated Crohn’s Disease Activity Index (CDAI) score at the time of presentation was greater than 300. Documented weight history reflected an around 30-pound weight loss (~17%; body mass index decrease from 24 kg/m^2^ to 19.8 kg/m^2^) over the previous year.

**Figure 1.**
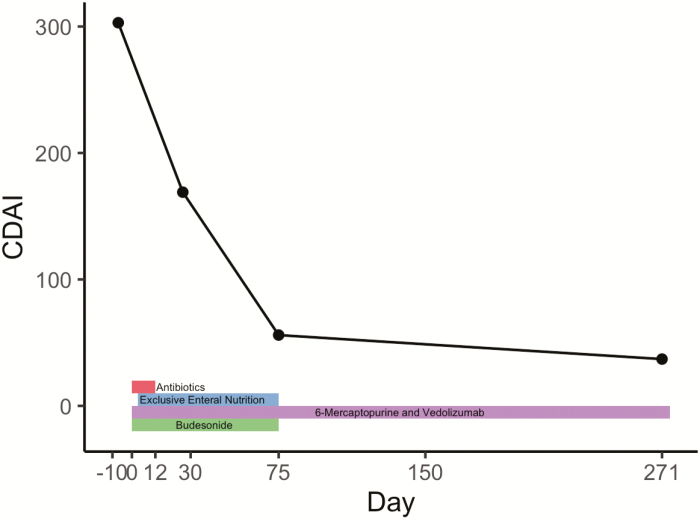
Timeline of interventions and change in CDAI score. This figure presents a timeline of therapies and changes in CDAI score over the course of therapy, including the exclusive semi-elemental formula diet intervention.

Following emergency department presentation, his IBD care team instituted the following plan of care: antibiotics (ciprofloxacin and metronidatzole) given fistulous disease, stop methotrexate and start 6-mercaptopurine, start vedolizumab, start budesonide, start exclusive EN diet utilizing elemental or semi-elemental formula, and colorectal surgery evaluation. A timeline of interventions is illustrated in Figure 1. The formula of choice for the exclusive EN diet was vanilla-flavored Peptamen 1.5. Due to logistical limitations obtaining the formula, the exclusive enteral formula diet was not started until day 3. No other oral intake was allowed, other than water (city water) for hydration. Antibiotics were stopped on day 12.

The patient was initiated on a regimen of 4 cartons of formula per day, which was estimated to provide 1500 kcal/day (24 kcal/kg) and 68 g protein/day (1.1 g/kg). This was subsequently increased to 6 cartons of formula per day [2250 kcal/day (37 kcal/kg) and 102 g protein/day (1.7 g/kg)] after 4 days due to continued weight loss. Additionally, after 4 days of the exclusive enteral formula diet, the patient began chewing sugar-free gum to help with a reported “funny taste” in his mouth.

Follow-up radiographic imaging (MR enterography) was obtained 34 days after original CT enterography (day 27), which demonstrated decreased inflammation, improved stricturing, and fistula healing. Nutritionally, he was able to maintain a stable weight on his exclusive enteral formula regimen. His CDAI score was estimated to be around 170 (remission <150).

The decision was made to continue the patient on exclusive EN until he was weaned off of budesonide. Ultimately, the patient remained on an exclusive enteral formula diet for 75 days. During this time he consistently reported 2–3 loose stools per day, which he anecdotally noted improved following course of antibiotics and later with the cessation of gum chewing. Bowel movements (BMs) did not become formed, however, until solid food was reintroduced. The patient pursued a conservative food reintroduction strategy per his preference. On day 95, the patient reported a daily intake of 4 cartons of Peptamen 1.5, 16 ounces chicken broth, 1–2 prepackaged Jell-O cups, a fruit smoothie (1 cup tropical fruit blend, 1 cup vanilla-flavored almond milk), 3 hard-boiled eggs, 1 tablespoon of almond butter, and an evening meal of 3 ounces lean meat or fish and a starch (white rice, potato, and gluten-free toast). At this time he reported 1–2 formed BMs per day and had an estimated CDAI score of around 60. The patient did not completely wean himself off of shakes until day 152 at which time he reported an average of 1 formed BM per day and a weight increase of around 10 pounds (7%; body mass index 21.2 kg/m^2^) since initiating the exclusive enteral formula diet regimen. He continued on 6-mercaptopurine and vedolizumab without recurrence of symptoms.

**Figure 2.**
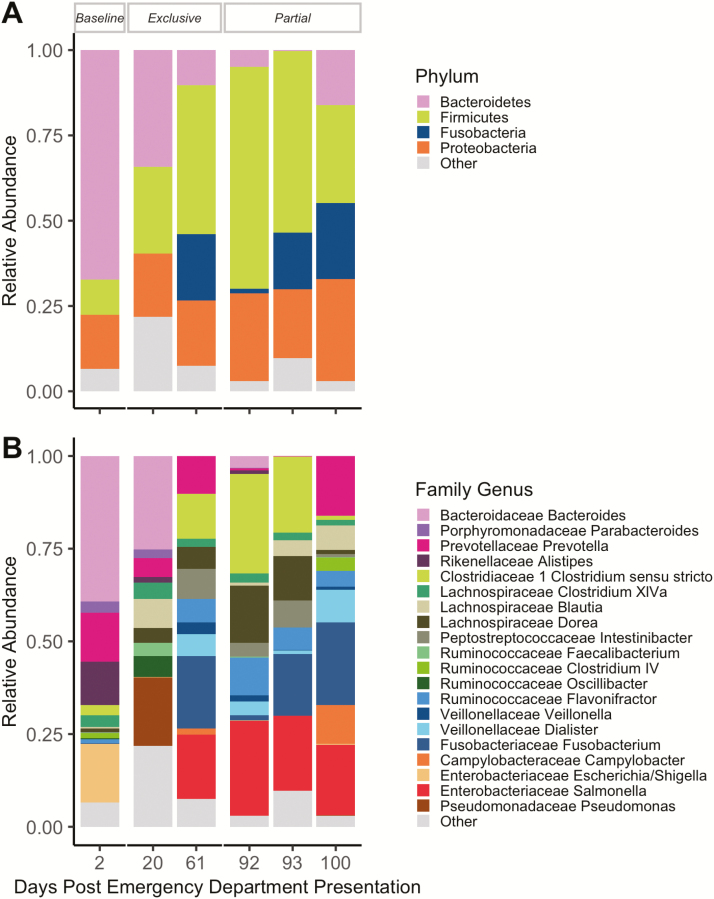
Microbiota compositional changes are associated with remission. The taxonomic composition is shown at the (A) Phyla and (B) Genera levels. Microbiota composition was assessed using 16S sequencing (V4 region), operational taxonomic units were clustered at 99% using USearch, aligned to a custom-curated Silva database with taxonomy annotations assigned by the RDP classifier. The “Baseline” period is the diet leading up to the initiation of exclusive enteral therapy. The “Exclusive” period is when the patient was following an exclusive enteral diet. The “Partial” period is when the patient was following a partial enteral diet.

Planned microbiota samples were collected throughout the course of EN through an IRB-approved stool collection protocol. At the phylum level (Fig. 2A), the observed increase in the relative abundance of Firmicutes and decreased relative abundance of Bacteroidetes was similar to previous findings in a pediatric population utilizing exclusive EN as an induction therapy.^7^ At the genus level, there was a notable decrease in the relative abundance of Enterobacteriaceae *Escherichia*/*Shigella* (Fig. 2B).

## DISCUSSION

The case presented here illustrates the possible utility of an exclusive enteral formula diet as an adjunct to medication to induce remission in an individual experiencing a CD flare. This case also underscores the precarious nutritional state associated with IBD flares and the positive impact an exclusive enteral formula diet can have on nutrition status.

The mechanism of action of exclusive EN therapy on inflammation in IBD is incompletely understood, but the gut microbiota has been proposed as a possible factor.^8^ In this individual, exclusive Peptamen 1.5, in conjunction with medication, supported a return to a microbiome state associated with remission. This patient also had a decrease in the relative abundance of Enterobacteriaceae *Escherichia*/*Shigella*, which is elevated in ileal mucosal biopsies of children with IBD although not previously seen in fecal samples.^9^ Although there were multiple variables in this case, and we cannot comment on causality, we hypothesize that alterations in the intestinal microbiota induced by EN decreased the stimulus for ongoing inflammation. Intestinal microbiota transplant is currently being studied as a therapy for IBD, and EN may be another way to modify the intestinal microbiota.

In summary, the implementation of exclusive EN therapy, in combination with medical therapy, is a feasible option for induction of remission in an adult patient with an acute CD flare.

## Notes

Funding: Nestlé funded part of this work.

Conflict of interest disclosure: L.M.T. is an independent contractor for Nestlé. E.S. has received travel support from Takeda. B.P.V. received research support from Takeda, Genentech, and Celgene and has received compensation from Janssen and AbbVie for speaking and advisory boards.
